# Supplementary material for: Impaired Antibody Response Is Associated with Histone-Release, Organ Dysfunction and Mortality in Critically Ill COVID-19 Patients
Source: J Clin Med. 2022 Jun 14;11(12):3419. doi: 10.3390/jcm11123419 (PMC9225468; doi:10.3390/jcm11123419)
Supplement: Supplementary file 1 [file jcm-11-03419-s001.zip › jcm-1693407-supplementary.pdf]

Supplementary material:

Methods

Cytokine analyses

The initial plasma sample obtained at the ICU for each patient was analyzed for 27 biomarkers with Bio-plex assay using a Luminex MagPix instrument (Bio-Rad Laboratories, Sundbyberg, Sweden) as listed: Interleukin (IL)-1β, IL-2, IL-4 to IL-10, IL-12, IL-13, IL-15, IL-17A, interferon gamma (IFN γ), interferon gamma-induced protein 10 (IP-10), monocyte chemotactic protein 1 (MCP-1, CCL2), macrophage inflammatory protein 1 alpha (MIP-1α), macrophage inflammatory protein 1 beta (MIP-1β), tumor necrosis factor (TNF), IL-1 receptor antagonist (IL-1ra), Regulated on Activation, Normal T Cell Expressed and Secreted (RANTES), Platelet-derived growth factor BB (PDGF-bb), Basic fibroblast growth factor (FGF), Granulocyte colony-stimulating factor (GM-CSF), Granulocyte-macrophage colony-stimulating factor (GM-CSF), Vascular Endothelial Growth Factor (VEGF), and eotaxin.

Complement analyses

Complement tests were performed as previously described.[1] In brief, concentrations of C3, C4, and factor B were quantified by nephelometry. The activation products C3a and sC5b9 were determined by ELISA using neoepitope-specific antibodies for capture. C3a by ELISA using monoclonal antibody (mAb) 4SD17.3 for capture and biotinylated polyclonal anti-C3a for detection and sC5b-9 by an in-house ELISA using anti-human neo-C9 mAb aE11 for capture and polyclonal anti-C5 for detection. C3d was analyzed by nephelometry after removal of high molecular weight forms of C3 by PEG precipitation.

COMPLEMENT TESTS									
**COMPLEMENT FACTORS**									
	**IgM**			**IgG**			**IgA**		
**Analyte**	**Pos n =103**	**Neg n = 12**	**P value**	**Pos n = 98**	**Neg n = 17**	**P value**	**Pos n =96**	**Neg n = 19**	**P value**
C4 (g/L) (0.13-0.32)	0.28 (0.20-0.40)	0.28 (0.23-0.41)	0.94	0.28 (0.21-0.41)	0.25 (0.18-0.30)	0.12	0.32 (0.21-0.41)	0.25 (0.19-0.29)	0.12
C3 (g/L) (0.67-1.29)	1.24 (0.97-1.46)	1.17 (0.88-1.54)	0.71	1.26 (0.98-1.47)	0.95 (0.88-1.20)	0.010	1.25 (0.98-1.48)	0.99 (0.84-1.28)	0.10
Factor B (g/L) (0.19-0.50)	0.56 (0.45-0.67)	0.56 (0.38-0.66)	0.38	0.57 (0.48-0.69)	0.42 (0.36-0.60)	0.006	0.58 (0.47-0.69)	0.43 (0.35-0.61)	0.006
**COMPLEMENT ACTIVATION PRODUCTS**									
C3a (ng/L) (< 200)	253 (174-407)	276 (160-888)	0.56	264 (173-429)	261 (160-319)	0.85	279 (175-437)	218 (159-273)	0.38
C3d (mg/L) (< 5.3)	7.0 (6.0-8.6)	7.6 (5.0-7.7)	0.68	7.1 (6.1-8.5)	7.5 (5.1-8.6)	0.63	7.2 (6.1-8.9)	6.6 (4.7-7.9)	0.20
sC5b9 (μg/L) (< 50)	145 (103-214)	138 (64-213)	0.48	154 (104-214)	109 (71-142)	0.11	162 (103-215)	119 (84-140)	0.10

Data are expressed as median (interquartile range, IQR). Statistically significant differences between groups marked in red. Antibody pos: Antibody positive before ICU discharge.

CLINICAL CHEMISTRY TESTS<br>ICU entry values and peak values									
	**IgM**			**IgG**			**IgA**		
**Analyte**	**Pos<br>n =103**	**Neg<br>n = 12**	**P value**	**Pos<br>n = 98**	**Neg<br>n = 17**	**P value**	**Pos<br>n =96**	**Neg<br>n = 19**	**P value**
**INFLAMMATION / COAGULATION**									
CRP day 1 (mg/L)	170 (118-237)	125 (63-199)	0.061	176 (120-237)	125 (63-165)	0.012	171 (118-237)	140 (64-195)	0.11
CRP peak value (mg/L)	286 (190-359)	270 (126-363)	0.58	290 (208-375)	193 (121-316)	0.018	287 (194-377)	233 (174-320)	0.14
IL-6 day 1 (ng/L)	115 (70-197)	86 (32-123)	0.099	115 (69-191)	87 (54-207)	0.52	118 (69-192)	87 (60-211)	0.47
IL-6 peak value (ng/L)	166 (83-324)	244 (86-276)	0.76	171 (85-341)	157 (74-276)	0.71	171 (86-304)	157 (77-290)	0.94
Fibrin, D-dimer day 1 (mg/L)	1.4 (0.9-2.2)	1.4 (0.5-2.8)	0.72	1.4 (0.9-2.2)	1.0 (0.6-2.8)	0.21	1.4 (0.9-2.3)	1.2 (0.7-2.3)	0.47
Fibrin, D-dimer peak value (mg/L)	3.3 (1.7-8.1)	3.7 (2.8-5.5)	0.47	3.4 (1.8-8.4)	2.6 (1.1-3.9)	0.071	3.4 (1.8-8.7)	2.8 (1.5-4.4)	0.19
Platelet count day 1 (*109/L)	215 (159-283)	154 (96-218)	0.090	212 (164-283)	154 (97-264)	0.084	205 (165-281)	156 (117-276)	0.25
Platelet count peak value (*109/L)	458 (337-646)	331 (109-527)	0.034	468 (345-648)	298 (160-434)	0.001	468 (353-650)	321 (171-448)	0.002
Platelet count min value (*109/L)	194 (145-255)	153 (87-205)	0.15	196 (150-250)	150 (97-247)	0.12	193 (149-247)	156 (109-268)	0.43
Lymphocyte count day 1 (*109/L)	0.90 (0.79-1.20)	1.20 (0.78-1.94)	0.20	0.90 (0.80-1.20)	1.05 (0.63-1.54)	0.62	0.95 (0.80-1.20)	0.95 (0.49-1.44)	0.76
Lymphocyte count peak value (*109/L)	1.70 (1.20-2.40)	2.20 (0.60-2.40)	0.91	1.80 (1.20-2.50)	1.30 (0.70-2.28)	0.054	1.80 (1.28-2.43)	1.30 (0.65-2.35)	0.10
Lymphocyte count min value (*109/L)	0.80 (0.60-1.08)	0.70 (0.50-1.30)	0.88	0.80 (0.60-1.10)	0.70 (0.53-1.08)	0.79	0.80 (0.60-1.10)	0.70 (0.45-1.40)	0.77

ORGAN DAMAGE BIOMARKERS

	IgM			IgG			IgA		
**Analyte**	**Pos n =103**	**Neg n = 12**	**P value**	**Pos n = 98**	**Neg n = 17**	**P value**	**Pos n =96**	**Neg n = 19**	**P value**
Creatinine day 1 (µmol/L)	79 (62-94)	98 (83-113)	0.034	79 (62-94)	101 (78-116)	0.017	79 (62-94)	105 (67-117)	0.011
Creatinine peak value (µmol/L)	95 (73-153)	115 (98-172)	0.11	95 (73-163)	110 (96-132)	0.36	96 (76-156)	110 (94-173)	0.48
Cystatin C day 1 (mg/L)	1.10 (0.94-1.30)	1.94 (1.43-2.25)	<0.0001	1.10 (0.95-1.32)	1.89 (1.10-2.15)	0.026	1.10 (0.89-1.32)	1.92 (1.12-2.29)	0.003
Cystatin C peak value (mg/L)	1.75 (1.28-2.87)	2.25 (1.94-3.39)	0.031	1.78 (1.30-3.08)	1.94 (1.63-2.65)	0.73	1.77 (1.30-3.03)	1.94 (1.44-3.08)	0.44
NT-proBNP day 1 (ng/L)	452 (196-1050)	310 (126-2208)	0.70	448 (196-968)	464 (106-1565)	0.78	318 (180-790)	1315 (345-2991)	0.017
NT-proBNP peak value (ng/L)	914 (412-2445)	4020 (670-8003)	0.025	938 (412-2735)	1545 (601-5143)	0.28	883 (392-2430)	2370 (827-4880)	0.021
Troponin I day 1 (ng/L)	15 (8-36)	30 (12-66)	0.15	18 (9-38)	13 (9-61)	0.93	15 (8-36)	23 (9-70)	0.28
Troponin I peak (ng/L)	23 (11-84)	108 (22-142)	0.040	24 (11-86)	33 (10-121)	0.63	23 (11-83)	53 (10-127)	0.26

BLOOD CELL COUNTS
ICU peak values

	IgM			IgG			IgA		
**Analyte**	**Pos n =103**	**Neg n = 12**	**P value**	**Pos n = 98**	**Neg n = 17**	**P value**	**Pos n =96**	**Neg n = 19**	**P value**
Leukocyte count peak value (*109/L)	13.4 (9.9-19.4)	15.3 (13.5-16.7)	0.56	13.7 (9.9-20.2)	13.8 (7.8-15.8)	0.28	13.5 (9.9-20.6)	14.1 (11.9-15.7)	0.55
Neutrophil count peak value (*109/L)	8.0 (5.7-10.6)	7.6 (4.2-13.8)	0.74	8.0 (5.7-11.1)	6.4 (3.6-10.8)	0.22	7.9 (5.7-11.1)	8.0 (5.2-9.4)	0.52
Eosinophil count peak value (*109/L)	0.16 (0.03-0.30)	0.03 (0.02-0.24)	0.30	0.16 (0.04-0.32)	0.03 (0.02-0.09)	0.014	0.17 (0.05-0.34)	0.03 (0.02-0.09)	0.002

Basophil count peak value (*109/L)	0.03 (0.02-0.07)	0.03 (0.01-0.06)	0.40	0.03 (0.02-0.07)	0.03 (0.01-0.04)	0.078	0.04 (0.02-0.07)	0.02 (0.01-0.03)	0.005
Monocyte count peak value (*109/L)	0.60 (0.40-1.10)	0.70 (0.25-1.30)	0.95	0.60 (0.40-1.10)	0.50 (0.28-1.05)	0.50	0.60 (0.40-1.10)	0.50 (0.28-1.03)	0.37

Data are expressed as median (interquartile range, IQR). Statistically significant ($p<0.05$) differences between groups are marked in red. Antibody pos: Antibody positive before ICU discharge. Day 1: First day after ICU admission. Peak value: Highest value during ICU admission. CRP: C-reactive protein. IL-6: Interleukine 6. NT-proBNP: NT-pro-brain natriuretic peptide.

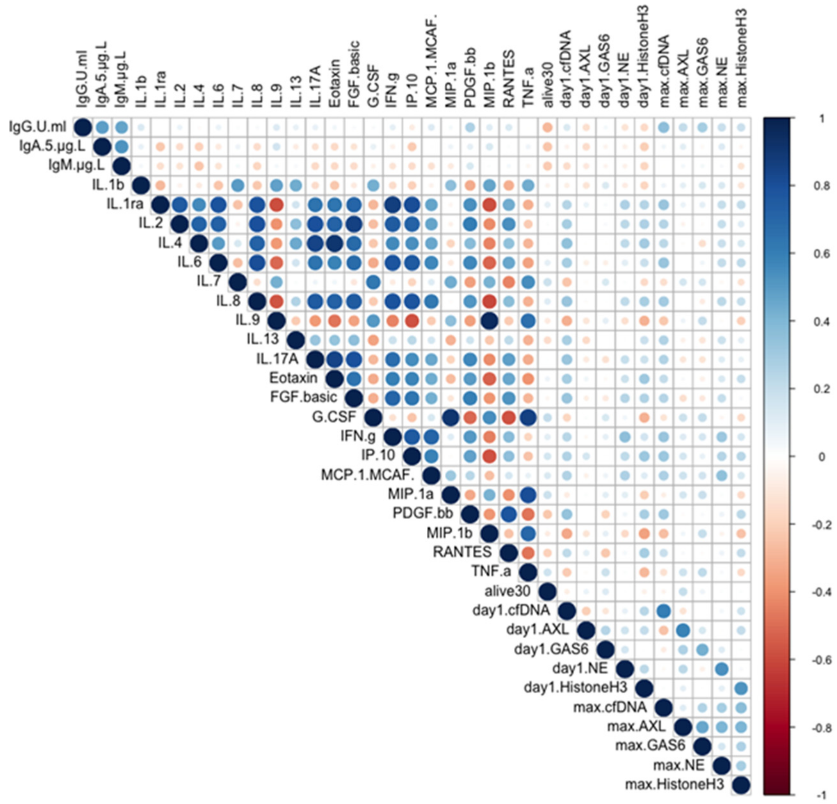

Correlations (Spearman-rank) of cytokine concentration in plasma at admission to ICU with concentration of IgM, IgA and IgG in plasma at the end of the stay in the ICU in critically ill COVID-19 patients. Blue colour indicates a positive correlation and red colour a negative corre-lation. The size of the symbol illustrates the strength of the correlation, a stronger relationship is indicated by a larger symbol. (IL = interleukin, TNF.a = tumour necrosis factor, IL-1ra = IL-1 re-ceptor antagonist, IP-10=interferon gamma-induced protein 10, G-CSF= granulocyte colony stimulating factor, MCP-1 = monocyte chemoattractant protein-1, MIP = Macrophage inflamma-tory protein, RANTES =Regulated upon Activation, Normal T Cell Expressed and Presumably Secreted, FGF = Fibroblast Growth Factor, PDGF = Platelet Derived Growth Factor and IFN =interferon).

References

[1] Lipcsey M, Persson B, Eriksson O, Blom AM, Fromell K, Hultström M, et al. The Outcome of Critically Ill COVID-19 Patients Is Linked to Thromboinflammation Dominated by the Kallikrein/Kinin System. Frontiers in immunology. 2021;12:627579.
